# Application of ADAPT-ITT: adapting an evidence-based HIV/STI mother-daughter prevention intervention for Black male caregivers and girls

**DOI:** 10.1186/s12889-023-16364-6

**Published:** 2023-07-26

**Authors:** Natasha Crooks, Alyssa Debra, Diamond Coleman, Wuraola Sosina, Randi Singer, Rohan Jeremiah, Betty Green, Waldo Johnson, Cleopatra Caldwell, Crystal Patil, Alicia K. Matthews, Geri Donenberg

**Affiliations:** 1 College of Nursing, University of Illinois Chicago, Chicago, IL USA; 2 College of Medicine, University of Illinois Chicago, Chicago, IL USA; 3 College of Medicine, University of Illinois Urbana Champaign, Champaign, IL USA; 4 Adler University, Chicago, IL USA; 5 Chicago Lawndale AMACHI Mentoring Program, Chicago, IL USA; 6 Crown Family School of Social Work, Policy, and Practice, University of Chicago, Chicago, IL USA; 7 School of Public Health, University of Michigan, Ann Arbor, MI USA; 8 School of Nursing, Columbia University, New York City, NY USA; 9 Center for Dissemination and Implementation Science, University of Illinois Chicago, Chicago, IL USA

**Keywords:** Adaption, Sexual health, Evidence-based, Black families, Community

## Abstract

**Background:**

Black girls are disproportionately impacted by HIV and sexually transmitted infections (STIs), underscoring the urgent need for innovative strategies to enhance the adoption and maintenance of HIV/STI prevention efforts. Historically, Black male caregivers have been left out of girls’ programming, and little guidance exists to inform intervention development for Black girls and their male caregivers. Engaging Black male caregivers in Black girls’ sexual and reproductive health may reduce sexual risk-taking and improve the sustainability of preventative behaviors.

**Objective:**

This paper describes the formative phases, processes, and methods used to adapt an evidence-based mother-daughter sexual and reproductive health intervention for Black girls 9–18 years old and their male caregivers.

**Methods:**

We used the ADAPT-ITT model to tailor IMARA for Black girls and their male caregivers. Diverse qualitative methods (interviews, focus groups, and theater testing) were used throughout the adaption process.

**Results:**

Findings support using the ADAPT-ITT model to tailor an evidence-based HIV/STI intervention for Black girls and their Black male caregivers. Findings highlight the importance of community engagement and the use of qualitative methods to demonstrate the acceptability and feasibility of the adapted intervention. Key lessons learned are reviewed.

**Conclusions:**

Adapting evidence-based interventions to incorporate Black girls and their Black male caregivers should be driven by a relevant theoretical framework that aligns with the target population(s). Adapting the intervention in partnership with the community has been shown to improve acceptability and feasibility as it is responsive to community needs. Using a systematic process like the ADAPT-ITT model will ensure that the new program is ready for efficacy trials.

## Introduction

Sexually transmitted infections (STIs) remain a significant public health problem for Black girls in the United States (US) [1, 2]. Each year 1 in 4 Black girls, 14–19 years old, acquires an STI, placing them at risk for poor sexual and reproductive health outcomes (SRH) (i.e., pelvic inflammatory disease, infertility, HIV/AIDS) [3]. In Chicago, STI rates are highest among 13–29-year-old Black females compared to other racial groups and represent 56% of new HIV diagnoses [4], making adolescence for Black girls an exceptionally vulnerable period [5]. These racial disparities require new and innovative strategies to reduce Black girls’ adverse SRH outcomes.

Individual, interpersonal, and structural factors increase Black girls’ vulnerability to adverse SRH outcomes [6]. At the individual level, early pubertal development is associated with significant SRH risks, including early sexual debut [7–10]. The difference between Black and white girls in early pubertal development has been associated with BMI and birthweight [11]. Unlike white girls who begin around age 12–14 years [10], Black girls start puberty as early as 8–9 years old [9, 12], and are more likely to initiate sexual contact before age 13 [12–14]. At an interpersonal level, early puberty has led to the treatment of Black girls as adult women. Black girls who look older and are perceived as “early bloomers” are typically labeled as “fast” and promiscuous by peers, adults, and society [13, 15]. This over-sexualization of Black girls by society (i.e., history, culture, and social media) can create distorted images of Black girls’ self-identity and treatment of others [16–19]. Additionally, through early sexualization and sexual contact, Black girls experience increased vulnerability to sexual violence and HIV/STI acquisition [20–22]. Despite the long-term health consequences of this inherently racist and sexist ethos, the adultification of Black girls has become an accepted social norm that threatens their SRH.

Adultification refers to the contextual, social, and developmental processes that prematurely and often inappropriately expose girls to adult knowledge and assume extensive adult roles and responsibilities [22]. Adultification reduces protection by adults during childhood due to the belief that Black girls’ cognitive development corresponds with their physical development [18]. At a structural level, the adultification of Black girls leaves them vulnerable to negative SRH outcomes. Furthermore, perpetuated assumptions that pubertal Black girls know about sex and are capable of giving consent may prevent girls from seeking health information or disclosing an experience of sexual violence [16, 21, 23].

Precipitous sexualization of Black girls often leads to adultification [22] perpetuating the silencing of Black girls and limiting their ability to disclose experiences of sexual violence [15]. Black girls report elevated rates of sexual violence; nearly 20% of Black women report rape in their lifetime [2], which is likely an underestimate because many Black girls may not recognize their first sexual encounters as violent or assault [24]. Sexual violence exposure increases risky sexual behavior, creates shame about early development, and reduces the utilization of sexual health resources and willingness to report victimization. To facilitate the protection of Black girls, a multilevel approach is needed to not only educate families and communities about ways to decrease girls’ vulnerability to negative SRH outcomes but also to foster self and collective empowerment [25]. Few HIV/STI-prevention family-based interventions target early adolescents and systematically engage multiple stakeholders (i.e., research experts and community organizations) to culturally tailor content for Black girls and their families at this developmental stage [26].

### Becoming a sexual Black woman framework

The application of behavioral theories and frameworks can provide a structure for systematically identifying determinants of change for intervention adaptation [27]. Traditional behavioral theories (i.e., social cognitive theory, the health belief model, self-efficacy theory, and the theory of planned behavior [28–47]) focus on individual-level risk and protective factors but lack attention to structural dynamics and the dyadic nature of social interactions that lead to sex, especially for adolescents [48, 49]. These theories were mainly developed for white populations and adults; few have been culturally adapted to meet the needs of Black girls [48, 49]. The Becoming a Sexual Black Woman (BSBW) framework, developed by the first author, and informed by the sexual experiences of Black girls and women, fills this gap [13, 16, 20, 50]. The BSBW framework emphasizes the impact of structural factors (i.e., racism, discrimination, sexual violence, stereotype messages, and adultification) which creates less protection of Black girls’ sexual development. More specifically, the BSBW framework articulates how structural factors (i.e., absence of protection and stereotyped messaging) influence Black female sexual development throughout life, from Girl to Grown, to Woman [16]. Sexual development is described as the process of physical, cognitive, and behavioral growth, including sexual maturation, socialization, identity development, and sociocultural influences [51]. The Girl phase (5–14 years old) has been described by Black girls and women as a period of naivety, vulnerability, and lacking control of bodies [20]. The Grown phase (11–17 years old) was characterized as a confusing time when girls are “figuring out” sexual identities [20]. The Woman phase (ages ≥ 18 years)) has been often associated with becoming a parent and a period of new insights, personal growth, and emotional strength [20].

Protection is conceptualized within BSBW as a strategy to prevent the early sexualization of Black girls and delay their progression to the next phase of sexual development until they were mature enough [16]. Protection was often described in the form of a person or “protector” having a profound influence on the sexual development of Black girls. Protectors were described as someone who were physically available to provide guidance and support for Black girls [16]. Although protectors were often female caregivers, they could be anyone, who cared about the girl’s well-being and tried to keep them physically and psychologically safe [16]. Black girls reported desiring such protection from their male caregivers [16]. Protectors may be absent or shift during the Girl and Grown phases, a time when stereotype messaging is particularly impactful. Stereotype messaging is visual images, words, or stories in social media, culture, and history that sexualize Black female bodies and expect sexual behavior [20]. Black girls are particularly vulnerable to historically (i.e., Jezebel) [17, 19] and culturally (i.e., fast-tailed girl) [13, 52] rooted sexualized stereotype messaging, leading to body shame and silence [20]. Social media amplifies girls’ risk for sexual violence and HIV/STI by perpetuating sexual objectification of Black bodies [16, 53]. Consequences of these messages are early sexual engagement, sexual trauma, and reluctance to report surviving sexual violence [20]. The BSBW framework guided the adaptation of IMARA, an existing evidence-based intervention (EBI), to address the SRH protection needs of Black girls by incorporating their Black male caregivers.

As indicated by the BSBW framework, Black girls have reported that parental protection mitigates the effects of stereotyped messaging, and they expressed wanting the same protections from Black male caregivers [13]. Including Black male caregivers in protecting Black girls in this framework will inform more inclusive SRH programming and reduce the negative impacts of structural factors [13]. Historically, Black women have protected and instilled protective strategies (e.g., setting rules, and boundaries, using labels such as “fast” or “fast-tailed” girls to discourage early sexual behavior, teaching girls to say no to inappropriate sexual advances and how to spot predators) [16]. Female caregivers often provide girls with information about their bodies, sex, and relationships more so than male caregivers. As a result, this study posits that protection should be expanded to include Black male caregivers.

### Black male caregiver impact on adolescent development

Families may be uniquely positioned to mitigate the racial disparities in STIs and sexual violence among Black girls. Most family-based HIV/STI prevention programs are designed for mothers or female caregivers [54, 55], and Black male caregivers are notably missing, yet the benefits of HIV/STI prevention may be strengthened by their involvement [56]. Historically, Black male caregivers have been prescribed distinct gendered social roles of being the provider and protector of families [56]. The term “male caregivers” is defined as men (e.g., fathers, grandfathers, uncles, brothers, cousins) who engage in the socialization process of Black girls. This socialization process includes caregiver communication, monitoring, discipline, and engaging in discussion about race and gender-based discrimination [57–59]. Leveraging these expectations may make Black male caregivers ideal collaborators in programs to reinforce the protection of Black girls’ SRH [56]. Additionally, single fathers are a growing public research priority, increasing from 1.7 million in 1990 to 3.3 million in 2020 [60]. Black male caregivers may be instrumental allies in preventing STIs and HIV in girls by addressing sexual violence, an epidemic in Black girls’ intimate partner relationships associated with risky sexual behavior. Research indicates that Black male caregivers help Black boys avoid violence and remain safe, and conversations Black male caregivers have with their boys about the pervasive threats of violence, emotional engagement, and their overall presence helps boys triumph over threats to their safety [61]. Black boys view Black male caregivers as credible and trustworthy messengers to communicate how to protect themselves [61] who have traversed a journey of adverse circumstances (i.e., racism, violence, police harassment, incarceration). Demonstrating better health outcomes for their sons, Black male caregivers are best suited to recommend strategies to navigate nuances when responding to tense and potentially violent interactions with gangs, police, and other law enforcement figures, [61]. In addition to violence, research related to Black male caregivers and boys shows that improvement in Black male caregivers-boy communication and relationship quality and increased involvement of Black male caregivers protects against boys’ risky sexual behavior (i.e., engaging in condomless sex) [56, 61–66].

These findings extend to girls; the presence of a male caregiver is related to later sexual debut and increased condom use among female adolescents [62–64, 67]. These data suggest that Black male caregivers can protect girls from sexual risk and sexually violent relationships through protective communication. Moreover, data indicate that Black girls are eager to receive SRH information from Black male caregivers noting their unique perspectives as “men” [59, 68, 69]. However, interventions must help Black male caregivers increase their comfort communicating with girls, identify barriers to effective communication, and provide practice to promote safer sex behaviors among Black girls [68]. We will target Black male caregivers because 1) evidence of improved health outcomes from research with Black boys and caregivers, 2) protection aligns with the perceived roles of male caregivers, and 3) protection has been identified as a moderator of sexual risk among Black girls.

### ADAPT-ITT model

The science of intervention adaptation has been significantly strengthened by models and guidelines on how to adapt programs and document these changes systematically [27]. The Assessment, Decision, Adaptation, Production, Topical experts-integration, Training, and Testing (ADAPT-ITT) model. ADAPT-ITT has been used to increase HIV/STI prevention efforts in key populations and outlines eight phases that include key stakeholders and quantitative and qualitative methods [70]. By adapting an already established evidence-based HIV intervention, researchers can save time and reduce the costs associated with creating an entirely new intervention, also allowing for faster delivery of crucial HIV/STI prevention information to populations with increased vulnerability [70]. Additionally, ADAPT-ITT allows for both the target population and staff to take an active role in developing a culturally safe study. One key benefit of this strategy is the early engagement of the community. This aids in increasing trust, especially in populations with a history of medical and research mistrust, and the likelihood of involvement in the intervention [71]. This article describes the application of the ADAPT-ITT model to a family-based sexual and reproductive health program initially designed for Black mothers and daughters, Informed Motivated Aware and Responsible about AIDS (IMARA) [54], for Black girls and their Black male caregivers. This article aims to describe a) the application of each phase of the ADAPT-ITT framework to the adaptation, b) the newly designed intervention, and c) lessons learned during the collaborative adaptation process.

## Methods

This formative study utilized the ADAPT-ITT model. Our findings align with 8 phases of ADAPT-ITT which include: 1) Assess, 2) Decision, 3) Administration, 4) Production, 5) Topic experts, 6) Integration, 7) Training, and 8) Testing [70]. Working with our research team, experts, participants, and community partners, the intervention was systematically adapted to reflect the needs and preferences of girls and male caregivers. Participant interviews and focus group data were audio recorded and transcribed. Participants completed survey data during theater testing. Qualitative descriptive analyses (i.e., thematic and rapid content analysis) were used to analyze interview, focus group, and theater testing data to adapt the IMARA curriculum to create IMARA for Black Male Caregivers and Girls Empowerment (IMAGE). The Institutional Review Board at the University of Illinois at Chicago approved all study procedures. Consent was collected for all participants. Additionally, written informed consent from the parents, or the legal guardian and assent was collected for participants under the age of 16 for all phases of this study. Table 1 outlines the adaption phases, questions, methodological decisions, and observations as described below.

**Table 1:** Application of ADAPT-ITT

**Phase**	**Methodological decisions**	**Results or observations**
**1. Assess** (Who is the new target population and why is it at risk of HIV?)	• Assessed Black girls’ HIV/STI risk profile • Examined theoretical approaches utilized with the target population • Conducted meetings with intervention experts for the target population	• The target population requires interventions to address sociocultural factors (i.e., lack of protection, stereotypes, and developmental needs) that impact Black girls • The intervention needed to address structural factors and barriers that limit Black men’s engagement in family-based interventions
**2. Decision** (What EBI is going to be selected and is it going to be adopted or adapted?)	• Conducted a scoping/literature review on HIV/STI intervention with Black girls • Selected IMARA intervention • Decided to adapt IMARA for Black male caregivers and girls, making content culturally and developmentally tailored for this population • Selected a theoretical developmentally sensitive framework (BSBW) • Selected LAMP as a partnering community organization	• Found gaps in family-based interventions, specifically engaging Black men • IMARA, an evidence-based intervention successfully reduced STIs among young Black girls • BSBW’s developmental approach recognizes how structural factors inform the sociocultural context of Black girls • The role of the organization is to ensure a “community-based” program and address the needs of the population. Community advisory was created within the partnered organization
**3. Administration** (What is the original EBI that needs to be adapted, and how should it be adapted?)	• Decided not to modify IMARA’s core elements • Conducted interviews with Black male caregivers and focus groups with the community advisory board • Produced draft 1 of IMAGE manual based on interview and focus group feedback	• Retention of primary intervention assures “evidenced-based” standards [72] • Important to have a community advisory board review content and timing • Important that the content and voices of Black male caregivers are highlighted and integrated • Theater testing allowed for real-time implementation of recruitment, modules, and retention
**4. Production** (How do you produce draft 1 and document adaptations to the EBI?)	• Produced draft 2 of the IMAGE manuals based on interview and focus group feedback	• Produced draft 2 of the adapted EBI • Developed an adaption plan • Balanced fidelity while maintaining the core elements of IMARA and incorporating the BSBW framework
**5. Topic Experts** (Who can help to adapt the EBI?)	• Identified experts knowledgeable about STI/HIV prevention, health disparities in Black men’s health, and girls’ development • Met with directors of partnering organizations	• Important to have both the community advisory board and intervention developers • Discussed the role, functionality, and costs of IMAGE within community organizations • Overall costs of a 6-week formative pilot were made
**6. Integration** (What is going to be included in the adapted EBI that is to be piloted?)	• Integrate feedback from participants and topical experts to produce draft 2 of the IMAGE manual • Integrated scales that measure new intervention content in the study survey	• Draft 2 completed • Reviewed drafts of survey measures and integrated them online
**7. Training** (Who needs to be trained?)	• Trained recruiters, facilitators, observers, and data staff to implement IMAGE • Produced draft 3 of the IMAGE manuals based on feedback from facilitators who also served as topic experts as they worked on the IMARA study	• Important to have feedback from facilitators to review content and timing • Draft 3 is completed and ready for pilot testing
**8. Testing** (Was the adaptation successful, and did it enhance short-term outcomes?)	• Submitted IRB • Administered a theater test with 6 dyads of Black male caregivers and girls to assess the acceptability and feasibility of IMAGE	• Obtained IRB review and approval for theater test • Set up for the pilot test and one month follow up

## Processes and results

### Phase 1: Assessment

#### Who is the new target population, and why are they at risk of HIV/STIs?

In Phase 1, we examined previous research and theory to identify the unique mechanisms linked to HIV/STI-risk behavior for Black girls [33, 54, 73, 74]. Preliminary qualitative data of the first author indicated Black girls as the target population, sociocultural conditions (i.e., early sexual development, adultification, and lack of protection) that place them at disproportionate risk of HIV/STIs, and the need for multilevel interventions to address their risk profiles [16, 20, 50].

A team consisting of graduate students in health-related fields (public health, medicine, and psychology) and experts in family-based HIV/STI prevention intervention, community engagement, and implementation science was created to ensure the intervention was systematically adapted and tailored to address developmental, cultural, and gender needs [16, 21, 75]. Most of our research team identified as Black (NC, AD, DC, WS, BG, RJ, WJ, CC) and had interest and expertise in health disparities and mental and sexual health.

### Phase 2: Decision

#### What EBI will be selected, and will it be adapted or adopted?

HIV/STI prevention EBIs for Black girls and women exist, but the effects are short-lived, and ongoing health disparities for Black girls require innovative approaches to strengthen the long-term effects. Well-known EBIs, such as Sisters Informing Sisters about Topics on AIDS (SISTA), Sisters Informing Healing Living and Empowering (SIHLE), and Women Involved in Life Learning from Other Women (WiLLOW), focus on modifying individual-level risky sexual behaviors, offer information about the cause, treatment, and prevention of HIV/STI, and counsel women about safer sex behavior and all of them were created for Black girls and women [29, 32, 76]. The positive impacts of these programs may be strengthened for Black girls by two factors: tailoring the program for families to address interpersonal and structural drivers to allow for reduction of Black girls’ sexual risk; and including more information for girls to better understand how knowledge, attitudes, messages, and values about sexuality impact the multidimensional process known as adolescence.

One family-based program, Informed, Motivated, Aware, and Responsible about AIDS (IMARA), encompasses both factors [54]. IMARA is an evidence-based psychosocial HIV/STI prevention program designed for Black mothers and daughters to address individual, social, and structural drivers of HIV/STI risk. IMARA leverages the mother-daughter dyad as a structural factor for girls to encourage behavior change by strengthening daughters’ perceptions of mothers as knowledgeable in sexual decision-making and shifting peer norms within the group in favor of prevention. IMARA is one of the only family-based programs focusing on the parent-adolescent relationship related to SRH and empowers parents as role models. It promotes Black values and the importance of family and parents as resources for prevention. Designed for mothers or female caregivers, the curriculum seeks to strengthen mother-daughter relationships and communication, enhance self-efficacy to use condoms, teach assertive communication, increase maternal monitoring, improve emotion regulation, and emphasize the role of social media and stereotype messaging on Black girls, all while creating pride in Black culture and gender empowerment. The IMARA curriculum consists of multiple scripted modules where mother-daughter dyads engage in activities to teach girls how to improve communication styles, engage in safe sex practices, protect themselves against domestic violence situations, and strengthen familial relationships. These highly interactive modules allow mother-daughter dyads to practice newly formed skills [54] actively. Modules include various activities (e.g., posters, video clips, condoms, social media images of celebrities, worksheets, and role-playing scripts) to allow for visual, auditory, and kinesthetic learning [54].

In Phase 2, Decision, our research team selected IMARA as the EBI to adapt for Black girls and male caregivers as it was a family-based intervention that was effective for Black girls in urban settings leveraging structural factors. In a 2-arm randomized controlled trial (RCT), girls who received IMARA demonstrated a 43% reduction in HIV/STI incidence at 12-month follow-up compared to girls who received a time-matched health promotion program [54]. IMARA underscores the impact of social and cultural drivers related to HIV/STI risk and can be adapted to include structural factors (i.e., incarceration, police brutality, and lack of protection) limiting Black male caregivers from protecting Black girls. The BSBW framework was selected to guide and address structural factors for this interventional project.

The first author had a pre-existing relationship with the Chicago Lawndale AMACHI Mentoring Program (LAMP), which was selected as our community partner [77]. LAMP provides opportunities for positive youth development for Black youth in the North Lawndale community by providing space to be themselves, learn, grow, and thrive in a community that is affected by disinvestments like poverty and crime [77]. LAMP leaders develop partnerships with organizations in the community such as the University of Illinois of Chicago, to prepare participating youth for opportunities promoting positive youth development. Youth connected with LAMP have been disproportionately impacted by incarceration and generational poverty [77]. LAMP’s innovative design elements include supporting and involving faith-based congregations, promoting strong personal relationships between youth and their mentors, and professional case management [77].

### Phase 3: Administration

#### What is in the original EBI that needs to be adapted, and how should it be adapted?

In the Administration phase, we conducted semi-structured interviews with 30 Black male caregivers (aged 22- 60) to assess how they conceptualize protecting Black girls. Most caregivers (80%) identified as biological fathers, 3.3% as non-biological fathers, 10% as stepfathers, 3.3% as adoptive fathers, and 3.3% as the guardian of their girls. Caregivers (66%) identified residential fathers (e.g., lived in the same residence with girls daily) and primary caretakers (e.g., spending every day with girls), and 33% identified as non-residential fathers (e.g., lived in a separate residence from girls) and secondary caretakers (spending time with girls. bi-weekly, once a month, etc.). Black male caregivers were asked: “What does being a Black male caregiver mean to you? and "What does protection mean? How do you protect Black girls" and "Can you tell me how you might or have engaged in conversations with your girl about sex and puberty?" Additionally, we asked male caregivers, "If you were creating a program for Black girls to protect their bodies better, what would you include?" Male caregivers were compensated $30 for participating in the interview. Findings from our interviews with Black male caregivers indicated they were interested in being involved in protecting Black girls’ sexual development but needed help building skills to comfortably communicate about SRH and to promote safer sex behaviors. Some of the topics Black male caregivers asked to discuss in programming were a lack of knowledge about female bodies and puberty, social media, body image, appropriate communication with girls about their bodies, and a lack of positive Black male role models. Black male caregivers indicated that they would feel safer and more comfortable talking about topics of girls’ sexual and reproductive health with Black female facilitators.

We then constructed a community advisory board (CAB) to help inform how best to create a culturally relevant family intervention. Our CAB was recruited through our partner organization and consisted of Black girls, and male and female caregivers attending LAMP. CAB members were Black girls (*n* = 10) between the ages of 9–17 years old, Black female caregivers (*n* = 7) between the ages of 32–45 years old, and male caregivers (*n* = 8) between the ages of 24–58 years old. We convened 2 CAB meetings with each group (girls, female, and male caregivers). We conducted a total of six focus groups with CAB members to collect initial feedback about the feasibility of engaging Black male caregivers in SRH programming, specifically, how we might make the IMARA curriculum more inclusive and engaging for Black male caregivers. Each focus group ranged from 3–6 members. Two trained Black female facilitators led each group. We provided participants with $50 compensation, dinner, and beverages. CAB focus groups were asked: “How should Black male caregivers be involved in Black girls’ puberty? Questions about their bodies and relationships?” and “Would you feel comfortable with how Black male caregivers would like to be involved?”. We then summarized the modules (topics) covered in IMARA and asked how to engage Black male caregivers in these discussions. We asked about the program’s acceptability, including the language used in modules, activities, program delivery, facilitator identity, and recruitment practices. We also knew the engagement of female caregivers would be critical in this research. Therefore, we asked about their level of comfort with a program for Black girls and their male caregivers, as well as the selection of male caregivers and how to engage them in the process safely. This information helped us to develop recruitment approaches and assent/consent procedures. Interviews and focus groups lasted approximately 60–90 min and were audio-recorded, transcribed, and analyzed for thematic content.

Thematic analysis method developed by Braun and Clarke [78] was used to analyze interview and focus group data. Thematic analysis was used to guide our analysis of our interviews, as we sought to better understand how Black male caregivers protect Black girls and what they needed to support Black girls’ SRH. Thematic analysis was also used to analyze focus group data, as we wanted to better understand the comfortability and processes around engaging Black male caregivers in SRH topics. We chose thematic analysis to develop more implicit themes and patterns from the data to either adapt or create new modules for the adapted curriculum. Thematic analysis was conducted by the research team of students (WS, AD, and DC) and a faculty expert (NC) in health disparities and qualitative methods. The first phase of this analysis included rereading the interview and focus group transcripts. The data were individually coded by each member of the team to generate initial codes [78]. The team met weekly to discuss discrepancies and reached a consensus on final codes to create themes [78]. Themes represented the experiences of multiple participants. The research team meetings allowed for discussion of analysis, authenticity of coding, and thematic development, to ensure validity of analysis. Once the themes were finalized, they were then reviewed with the IMARA program developer to produce the initial draft of the curriculum.

### Phase 4: Production

#### How are adaptions of the EBI produced, drafted, and documented?

In Phase 4, Production, we utilized feedback from the interviews with 30 Black male caregivers and 25 CAB member focus groups on IMARA, which resulted in the initial draft of the new program IMARA for Black Male Caregivers and Girls Empowerment (IMAGE). Consistent with EBI adaptation literature, core components were preserved to maintain the fidelity of the original intervention while refining the new program to ensure relevancy to the target population [79]. We worked closely with the developer of IMARA to ensure core elements were retained: 1) HIV/STI cognition and skills, 2) mental health/emotion regulation, 3) effective communication, 4) parental monitoring, and 5) partner/relationship characteristics and power dynamics. However, we added content about the impact of structural factors – including stereotype messaging, sexual objectification, and intimate partner violence, adapted the role-plays and scenarios to include Black male caregivers, and integrated the BSBW theoretical underpinnings to highlight key constructs of Black female sexual development (i.e., early sexual development, lack of protection, and stereotype messaging).

### Content-related adaptions for IMAGE

After evaluating the IMARA curriculum, a thematic analysis was conducted, and five themes emerged from the interviews and focus groups [78]. These themes included: 1) lack of knowledge about female adolescent development, 2) structural factors (i.e., incarceration, police brutality, and lack of protection) impeding Black men from protecting Black girls, 3) toxic masculinity and lack of positive Black role modeling, 4) body positivity, and 5) challenges of Black male caregiver-girl communication about SRH. The themes, supporting quotes from Black male caregivers, and modifications made to the curriculum are in Table 2. New elements to the program were added that included new materials, activities, and content to enhance IMAGE’s relevance for Black male caregivers. The curriculum refers to the intervention manual, and modules are the topics in the curriculum.

**Table 2:** New themes produced, illustrative quotes, and modifications made

**Themes**	**Quotes from Black male caregivers**	**Modifications made**
Lack of Knowledge of Adolescent Development	“The female body, there should be information about breaking it down on what you have inside of your body, and what does this, and what does that” (22 years old)	We begin the curriculum with the BSBW framework and created two new modules “Your Body Your Birth Control," for girls and “Adolescent Jeopardy" to address the lack of knowledge about adolescent development for both girls and male caregivers
Structural factors (i.e., sexual violence)	“Sex, rape, how to protect yourself, how to carry yourself as a woman, how to be aware of your surroundings.” (25 years old)	We added the “Black male Caregivers Challenge Absenteeism and Protection, module for both girls and male caregivers to address components of structural factors (i.e., mass incarceration, police brutality, and lack of economic opportunities) that often reduces opportunities for Black male caregivers to protect and support Black girls. We added “Mental Blocks” for caregivers to discuss mental health
Toxic Masculinity and Lack of Positive Black Male Role Models	“[Men need to] quit looking at women like a piece of meat. Because like I said, I did that, and I realized that they're not. They have minds and souls of their own. It comes down to, "Treat that woman the way you want to be treated." (56 years old) “Positive, healthy adults, where they're able to talk with and process with, and establishing healthy relationships, that can help coach and guide and mentor men through difficult periods, time in their life.” (43 years old)	We addressed transgenerational behavior and toxic masculinity among Black men in the creation of a new module, “Black Male Caregivers Challenge Toxic Masculinity.” We discuss the lack of positive Black role models in the “Your Partner Your Choices,” module and discuss how characteristics of partners may be reflected in and influence the choices and behaviors their girls exhibit
Body Positivity	“For one, plastic surgeries. You need to first love your body first, not change it. Plastic surgery. We should make awareness of how dangerous plastic surgery is on a woman. We should bring up self-esteem” (27 years old)	We encourage body positivity in a new module, “Young, Black and Female” for girls to identify positive attributes of being a Black girl. We created two new modules “Black female stereotypes," and “Guess Who has an STI", for both girls and male caregivers to address stereotypes and assumptions made about Black girls and women
Challenges of BMC and Girl communication about SRH	“I'd let them [girls] know it's okay to talk to me about it if you need to, to know that you should be able to feel comfortable enough to talk to me. Let them know it is okay. No matter what our grandparents and our parents said. If you feel comfortable enough to have the conversation with any male, father figure especially, you should have that conversation.” (38 years old)	We have multiple modules to address communication, however, we created “The Big Talk: Girls Talking to Black male caregivers about Sex” to reduce the discomfort of discussing sexual health content among Black male caregivers and girls

#### Lack of knowledge about female adolescent development

To address this theme in the curriculum, we begin Day 1 by grounding the IMAGE program with the BSBW framework, which describes Black girls’ sexual developmental process, phases of sexual development, characteristics of early sexual and physical development, and adultification of Black girls’ bodies. Utilizing the BSBW framework provided participants insight into protecting Black girls and why it is necessary. This model also provided a foundation for how stereotyped messaging and protection influence Black girls’ sexual development. More specifically for Black girls, we included new modules that help address concerns about their physical, mental, and emotional development during puberty. In the module “Your Body Your Birth Control,” girls are educated on the different kinds of birth control and practice choosing effective birth control methods for characters in different scenarios to empower them in making their own reproductive decisions. We added interactive activities such as “Adolescent Jeopardy” for both Black male caregivers and girls to teach them about female puberty and menstruation.

#### Structural factors

The BSBW framework also helps us introduce the impacts of structural factors (i.e., incarceration, police brutality, and lack of protection) on Black girls’ developmental process. We added two modules, the first, “Black male caregivers Challenge Absenteeism and Protection," speaks explicitly to the topic of incarceration and how that limits the protection of Black girls. In this module, we described protective strategies if Black male caregivers cannot be physically present or live near their girls. For Black male caregivers, we added modules to improve their knowledge about their influence as caretakers, initiating discussions on mental health, and the importance of protecting Black girls. In the second module “Mental Blocks," we destigmatize conversations around mental health, acknowledge Black male caregivers’ struggles using Jenga blocks, and allow caregivers to have an open discussion about their burdens, along with providing healthy and alternative health strategies. This was crucial to highlight the importance of mental health conversations and allow Black male caregivers to have a safe space to discuss challenges among other Black men.

#### Toxic masculinity and lack of positive Black male role models

To address transgenerational behaviors and toxic masculinity related to being a Black man, we created a module, “Black Male Caregivers Challenge Toxic Masculinity," to debunk what it means to be a Black man and to address cultural norms associated with being a Black man. Participants do an activity where Black male caregivers create a gingerbread person and the positive attributes, they associate with being a Black man. In another module, “Your Partner Your Choices," we discuss how characteristics of partners Black male caregivers have may be reflected in and influence the choices and behaviors their girls exhibit. We also discuss the lack of positive Black male role models in the media associated with the assault of Black girls and women (e.g., Bill Cosby and R. Kelly). Ensuring that Black male caregivers learn protective strategies was critically important to the success of this intervention. Some of these strategies included: calling out creepy or predatory male behavior, having difficult conversations with family members or men in the community, talking to girls about protecting their sexual health, and calling out girls’ ages and labeling them as minors.

#### Body positivity

To address body positivity, we added the module “Young, Black, and Female” Girls identify positive traits associated with Black females to instill pride in themselves and others within their community. For both Black male caregivers and girls, we added the module “Black female stereotypes," which included visual activities with images of famous Black women in Black culture historically and currently in the media to promote body positivity. In this module, we do the “What you See vs. What is True” activity and address myths about Black women’s bodies and risks associated with increasingly common surgeries such as Brazilian Butt Lifts. We also discuss what healthy Black female bodies look like and that they come in all different forms, shapes, and sizes. In the module “Guess Who has an STI?”, Black male caregivers and girls, within their groups, had the chance to learn about vaginal discharge, ways to identify and treat sexual and non-sexual (e.g., yeast infections and bacterial vaginosis) infections, the risks of profiling potential partners based on image (i.e., assuming someone has an infection based on how they look rather than through HIV/STI testing), ways to protect themselves from infections (i.e., abstinence or condoms), repercussions of untreated infections (i.e., pelvic inflammatory disease and infertility), and when to seek treatment from a medical professional.

#### Black male caregiver-girl communication about SRH

To address the challenges of Black male caregiver-girl communication about SRH, a new module, “The Big Talk: Girls Talking to Black male caregivers about Sex,” was created. The module included important topics to discuss with Black male caregivers (e.g., sex, relationships, bullying, fighting, body image, sexuality); identify reasons why they may avoid these conversations; and identify trusted adults, male or female, they feel comfortable discussing these topics with, to encourage increased communication. The original IMARA curriculum explores different types of communication, including assertive vs. passive and role-playing effective girl-caregiver communication.

### Phase 5 & 6: Topic experts and integration

#### Who can help adapt the EBI, and what additional content should be included?

In Phase 5, content experts (*n* = 6) were consulted to assist in curriculum development in areas where the adaptation team lacked expertise in Black male health, structural racism, and HIV/STI interventions engaging male caregivers and youth. We invited individuals with expertise on Black men and structural racism to review the adapted curriculum and provide feedback. Experts suggested additional activities to support Black male mental health and address toxic masculiniy and stereotyping. Informed by previously successful interventions with Black men, experts went through draft 1 of the IMAGE curriculum and commented on ideas for activities to better engage Black men. They helped to ensure that critical content and considerations were included in the adapted curriculum. Experts encouraged us to include a module about Black male mental health and resources in the IMAGE curriculum, as discussing structural factors may be challenging. Feedback from the topic experts guided us in improving the IMAGE curriculum, which was better tailored for Black male caregivers and girls. Additionally, experts helped us integrate scales that measure new intervention content in the study survey. These were referred to as baseline measures and were collected during Day 1 of the theater test. In Phase 6, we integrated the input of experts to create the second draft of the IMAGE manual.

### Phase 7: Training

#### Who needs to be trained?

Facilitators (i.e., individuals who deliver the intervention for dyads) were a combination of experienced IMARA group leaders and individuals with training as health educators. Facilitators who previously worked for IMARA brought comfort and familiarity with Black girls and the IMARA curriculum. New facilitators were sought to increase staff availability and allow for additional perspectives when reviewing modules. Staff had different levels of experience working with adolescents, Black men, and group facilitation, and many had previously worked in fields such as medicine, public health, social work, and psychology. To support intervention fidelity, three in-person 8-h training workshops were held to ensure the competency of facilitators. Training sessions included four hours of reviewing modules for Black girls and male caregivers to ensure that all facilitators felt comfortable working with either group. Reviewing modules involved having staff read and act out activities with the corresponding material. After each module, feedback was requested from each staff member to improve content and delivery. The facilitator training also included the practice of utilizing developmentally appropriate for young girls. For example, our newly adapted modules included utilizing the language of participants that we gathered from focus groups and interviews, such as “grown” to describe their developmental phase or “sneaky link” which refers to having a secret affair. Facilitators were also instructed to adjust language based on the comfortability and participation of girls. Additionally, since our population may include individuals who are not cisgender or heterosexual, it was essential to train facilitators on all aspects of gender and sexuality (i.e., gender and sexuality spectrum, appropriate and respectful terminology, and what to do in the event offensive language is used). Facilitators and staff were a trained to intentionally create a safe space when delivering the intervention and challenge participants’ perspectives about what may cause tension within the group. Some of the IMAGE facilitators previously worked as facilitators for the IMARA study, and they provided feedback on new topic integration, the pacing of modules, and flow. This resulted in the third draft of the IMAGE curriculum.

During the training and theater testing facilitators completed evaluations of one another, including each module’s adherence and competence. Facilitators completed fidelity measures, that included observation of IMAGE sessions and feedback for new facilitators. The senior facilitators determined mastery of the material and completed observer ratings of each session to verify that the intervention was delivered as planned and the quality of the delivery was maintained. Questions were focused on if facilitators: followed the script for the session, explained each activity, demonstrated each activity, provided corrective feedback for incorrect responses, maintained quick pacing throughout the lesson, were open and non-judgmental, and the comfortability of facilitators with participants.

### Phase 8: Testing

#### Was the adaptation feasible and acceptable to the target population?

After adequately training the staff, we moved to Phase 8 and conducted a theater test of IMAGE using the new content developed for dyads of Black male caregivers and girls.

### Theater testing

We recruited girls and male caregivers attending LAMP into a theater test of IMAGE. Theater testing is a “pretesting methodology” that is commonly used to test interventions with the intended audience and how they respond to the intervention [70]. At the end of the intervention, participants complete a questionnaire to answer questions to gauge their reactions to the intervention. A strength of theater testing is the opportunity to obtain reactions to messages, concepts, and materials in a relatively short period that closely resembles the intervention [70].

We recruited by placing flyers at LAMP and active recruitment at community-based outreach events. We theater-tested the IMAGE curriculum with six dyads (*N* = 12). Each dyad consisted of one Black male caregiver to one girl. Black girls were 11–15 years (M = 13), and male caregivers were 25- 65 years old (M = 36). 83% of the male caregivers were < 25 years old, 50% were biological fathers, 33% were primary family members (uncles and grandfathers), and 67% were single or never married.

Consistent with the theater testing approach described by Wingood and DiClemente [70], newly recruited Black male caregivers and girls participated in each module, administered by trained Black female facilitators. Participants provided feedback on each module via a 4-item evaluation about the acceptability and included the open-ended question “What changes or suggestions do you have to improve the content?” Experienced IMARA facilitators delivered the curriculum, allowing a smoother transition from the IMARA to the IMAGE curriculum. IMAGE was delivered over two consecutive days, Saturday, and Sunday, at LAMP, consistent with IMARAs two-day RCT. The theater test allowed for evaluation of feasibility (i.e., can enough BMCs and girls be recruited and enrolled to participate, and could the intervention be fully delivered within two 6-8 hour days), acceptability (i.e., did Black male caregivers and girls feel they benefited by participating), and tolerability (i.e., could facilitators deliver the necessary content and did Black male caregivers and girls remain engaged). Our community partner, LAMP, selected participants who had never participated in IMARA. The theater test was conducted at the organization (LAMP) because of the proximity to the target population. Participants were compensated a total of $125 for their participation in IMAGE. Both girls and male caregivers received $60 on Day 1 after baseline measures were completed and $65 on Day 2 for the post-evaluation survey. Consistent with IMARA, four facilitators, two with the Black male caregiver group and two with the Black girl group, delivered the intervention. The intervention ran for 6 h each day. After each module, feedback was collected from participants via survey evaluation.

We collected acceptability ratings from evaluation forms each participant completed after each module. Overall, male caregivers and girls rated each module highly, with mean scores ranging from 3.6 to 4.0 (scale: 1 = strongly disagree, 4 = strongly agree). The overall acceptability of IMAGE was 3.8/4.0 for both male caregivers and girls. All acceptability questions scored a 3 (somewhat agree) or higher. Both girls and male caregivers were actively engaged and satisfied with the program. In open-ended responses, girls and male caregivers reported learning new information about HIV/AIDS, how to communicate more effectively with each other and their sexual partners and having greater comfort talking to each other about sex and condoms. We had 100% retention for both days of the IMAGE intervention. Baseline survey measures took about an hour and 15 min to complete.

Additionally, each group (girls and male caregivers) had a program observer to provide feedback on the improvement of content and delivery by facilitators and the program. Assessors were faculty and were selected by the PI. The observer took notes to capture verbal and non-verbal nuance. The two observers rated the following components of the theater testing workshop (5-item scale: 0 = poor, 4 = very well): how smoothly the sessions were administered (mean score = 3.6); how engaged participants were (mean score = 3.8); how comfortable participants were with the material (mean score = 3.4); and how comfortable participants were with the facilitators (mean score = 3.6). Overall, comments from participants indicated that male caregivers and girls found the IMAGE activities informative, effective, and fun.

After the theater test, a rapid content analysis by Hsieh and Shannon [80] was used to analyze theater testing data. Rapid content analysis was used to interpret the meaning from the qualitative data, with a directed approach starting from prior research findings as guidance for the initial codes (previously identified themes in phase 3) [80]. Rapid qualitative analysis involved eliminating transcription and summarizing data by theme or topic, rather than in-depth manual coding of transcripts [81]. Rapid qualitative analysis was chosen as the results were needed to quickly develop or modify implementation strategies for the IMAGE pilot. The coding team (NC and AD) achieved an inter-rater reliability of 0.90 across all codes. The responses from the observers and participants’ open-ended survey questions were employed to identify additional themes in the dataset to further refine the IMAGE curriculum [80]. This analysis revealed participants wanted more content and resources about birth control, discussion about age and consent, and to make more interactive modules. The participant feedback also revealed the need to reorganize and combine modules for better flow. After modifications, we created a finalized IMAGE curriculum ready to pilot in Table 3.

**Table 3:** IMAGE curriculum: themes, modules, and target participant

**Theme**	**IMAGE Modules**	**Target**
Lack of Knowledge of Adolescent Development	**Becoming a Sexual Black Woman overview** **Adolescent Jeopardy: What is true vs what you see (Body positivity and assumptions)** **Your body, Your birth control** Virus carrier handshake; High, moderate, low risk situations LIPSTICK (acronym for condom use steps) Alcohol, Drugs, & Sex PrEP; female condom use KISS – Keep It Simple Sister (Comebacks to pressure lines)	J BMC, G G BMC, G BMC, G J BMC, G G
Structural factors	Feelings as triggers for risk behavior; Feeling thermometer; Healthy coping Links between mental health and risk behavior Observed BMC-girl conflict discussion about incarceration, absenteeism, and protection **Mental Blocks** **BMC challenges absenteeism and incarceration**	BMC, G BMC, G J BMC J
Toxic Masculinity & Lack of Positive Black Male Role Models	Get to Know You Game; Reverse role-plays Compliments for free Creating personalized monitoring plans Sexting and cyberbullying **Your partner, your choices (Black male role modeling)** Music, movies, and TV Images that portray Black men and women (positive and negative) **Young, Black, and female-listing positive characteristics, stereotypes** **BMC challenges toxic masculinity**	J J BMC, G BMC BMC, G G G BMC
Body positivity	**Black female stereotypes (historical, media, and cultural)** **Guess who has an STI?** Pieces and parts of relationships, gender-role stereotypes Healthy versus unhealthy relationships, concurrent partnerships Choosing healthy relationships; What does abuse look like? Partner selection/types (casual, serious) & implications for HIV risk	BMC, G, BMC, G BMC, G BMC, G BMC, G
Challenges of BMC and Girl communication about SRH	Passive, aggressive, and assertive communication w/ role plays Observed BMC-girl conflict discussion about protection; BMC-girl values discussion Rephrase it-game – Using I-statements Role-play different types of communication **The Big Talk BMC-girl values about sex discussion**	BMC, G, J J BMC, G J J

Bold = new modules

*J* Joint, *BMC* Black male caregivers, *G* Girls

## Discussion

This paper describes the systematic adaption of a family-based intervention for Black mothers and daughters to an evidence-based sexual health intervention for Black girls and male caregivers. The systematic approach of the ADAPT-ITT framework was clear and effective in phases 1 through 8. Black male caregivers may be the missing ingredient to amplify the positive effects of SRH programming for Black girls. Engaging Black male caregivers in this work can increase protective effects among Black girls. Early and repeated inclusion of the target population, the community advisory board, was essential in developing IMAGE. Results underscore the importance of the application of ADAPT-ITT to adapt EBI. At each stage of the adaptation, feedback on content (e.g., language and topics), materials (e.g., posters and handouts), and delivery (e.g., length and presentation of each module) were collected by CABs, topic experts, facilitators, and participants. In each section below, we document feedback given and the adaptations made. In summary, we have 4 lessons learned when considering engaging Black male caregivers in sexual and reproductive health interventions designed to protect Black girls.

### Lessons learned

#### Lesson 1: Consider the selection of theoretical frameworks and how they align with the target population(s) and study aims

We utilized a “new” framework, BSBW, that has not yet been used in intervention development. However, it was critical that the framework aligns with the intervention’s content and aims and include the developmental perspective of the target population(s). The framework was grounded in the voices and perspectives of Black girls and women, with core constructs of sexual development, stereotype messaging, and protection rooted in Black culture, ensuring it was the right fit for the intervention [20]. Although the framework is not for Black men, it speaks to the aim of the study, which is to protect Black girls’ SRH. Many adapted interventions typically do not introduce another theoretical framework but rather utilize the theoretical framework their selected EBI utilized. This highlights the unique development of IMAGE as it introduces important aspects to assist the target population focused on in the original EBI while also retaining its core elements.

#### Lesson 2: Ensure content is not only culturally tailored but sensitive to gender when targeting two different populations

This was critically important as social norms, roles, and expectations vary by gender within the Black community. We selected images of women and men for module activities that both Black girls and male caregivers would know. We also had to be cognizant of generational differences in language. We had to make sure facilitators were trained to create a safe space and feel comfortable calling out or challenging participants’ perspectives. It was also important to provide participants with a space to talk about their feelings, norms, and beliefs about sexuality, which our content naturally created. Some of our topic content was intentionally controversial (i.e., discussions about Bill Cosby and R. Kelly as they are culturally seen as “the beloved fathers/pillars of the Black community”) to invite these conversations. Since these conversations were emotionally charged, consistent rotation of staff, who were available, was done to ease the emotional burden of intervention delivery.

#### Lesson 3: Engaging community stakeholders is critical to assessing acceptability and feasibility

Even during a pandemic, we knew that the engagement of CABs was critical to get buy-in and assess the acceptability and feasibility of IMAGE. Utilizing the ADAPT-ITT framework allowed us to engage in community-based research that directly involved the community. CABs allowed us to gauge participants’ comfort in participating during the pandemic (i.e., was Zoom or being in person with masks preferable when conducting focus groups, interviews, theater testing, and pilot testing). Engaging communities, experts, and the actual individuals delivering the intervention are critical. Feedback from all stakeholders is needed to get participants’ buy-in and deliver culturally informed content.

#### Lesson 4: ADAPT-ITT process can provide rigorous adaptation of existing protocols to new populations

The ADAPT-ITT model may be helpful in the development of interventions as many funding agencies encourage investigators to adapt existing EBIs. This process helps investigators plan and carefully evaluate procedures needed to implement the program on a larger scale, in our case, with more community partners and with a new population (male caregivers). The components of the ADAPT-IT model (i.e., theater testing) help to adapt the intervention systematically and leads directly to the piloting of the intervention, which can be helpful for early/new investigators. This process provided us with real-world-timing of activities and procedures as we witnessed that survey measures on Day 1 took about one hour and 15 min to complete, which encroached on the timing of the curriculum. The surveys also took a lot of energy out of participants, which led to them being less engaged in activities on Day 1. We will integrate this into the pilot and have the participants complete the baseline measures online before Day 1 of the intervention.

### Limitations

Fidelity is a limitation as it is challenging to measure and retain core constructs of original intervention (IMARA) when tailoring for different genders. Other limitations include the geographical location as it took place in a large urban city. The population included Black urban families who may not translate across cultures or in rural cities. This study should have larger sample sizes as our theater test was conducted with only 6 dyads. There may be variations in IMAGE delivery as facilitators have different facilitation styles. The inclusion of the BSBW as a second and novel framework in addition to the ADAPT-ITT Model was also challenging. As the BSBW framework was used as an implementation science framework that informed the need for tailoring an existing EBI to incorporate structural factors.

### Future research

Future research should consider the time the adaption process takes and the importance of theater testing. Due to the high retention rate and positive participant reviews, a pilot study is needed to include more participants. The next step is to pilot the intervention and conduct an efficacy trial of IMAGE to examine the impact on Black girls’ SRH outcomes.

## Conclusion

This article offers insight into applying the ADAPT-ITT model to an EBI targeting two vulnerable populations (Black girls and male caregivers). We demonstrate how this model helped retain core elements of IMARA while making it current, gender and culturally tailored, and accessible for the target populations. Efforts should be made for other programs to be created to look at including Black male caregivers in the protection of Black girls SRH. This paper represents an initial step toward engaging Black male caregivers in family-based HIV/STI prevention interventions.

## Data Availability

Availability of data and materials are available upon request from Dr. Natasha Crooks ncrooks@uic.edu.
